# Randomized clinical trial on the clinical effects of a toothpaste containing extra virgin olive oil, xylitol, and betaine in gingivitis

**DOI:** 10.1038/s41598-023-33521-4

**Published:** 2023-04-18

**Authors:** Alejandro Rodríguez-Agurto, Manuel Bravo, Antonio Magán-Fernandez, Ana López-Toruño, Ricardo Muñoz, Joaquín Ferrer, Francisco Mesa

**Affiliations:** 1 Department of Periodontics, School of Dentistry, University of Granada, Granada, Spain; 2 Department of Preventive and Community Dentistry, University of Granada, Granada, Spain; 3 Odontólogo, Centro de Salud de Loja, Unidad de Salud Bucodental Distrito Metropolitano, Servicio Andaluz de Salud, Granada, Spain; 4 Odontólogo, Centro de Salud de Huétor-Tajar, Unidad de Salud Bucodental Distrito Metropolitano, Servicio Andaluz de Salud, Granada, Spain; 5 Facultad de Odontología, Universidad de Granada, Campus de Cartuja s/n, 18071 Granada, Spain

**Keywords:** Dentistry, Dental pharmacology, Periodontics, Preventive dentistry

## Abstract

To determine the effects on gingival bleeding, dental biofilm, and salivary flow and pH in patients with gingivitis of using toothpaste with extra-virgin olive oil (EVOO), xylitol, and betaine in comparison to a placebo or commercial toothpaste. This controlled, double blinded, and multicenter randomized clinical trial included patients with gingivitis randomly assigned to one of three groups: test group (EVOO, xylitol, and betaine toothpaste), control group 1 (placebo toothpaste), or control group 2 (commercial toothpaste). Percentage supragingival biofilm and gingival bleeding were evaluated at baseline (T0), 2 months (T2), and 4 months (T4), measuring non-stimulated salivary flow and salivary pH. Comparisons were performed between and within groups. The final study sample comprised 20 in the test group, 21 in control group 1, and 20 in control group 2. In comparison to control group 1, the test group showed significantly greater decreases in gingival bleeding between T4 and T0 (p = 0.02) and in biofilm between T2 and T0 (p = 0.02) and between T4 and T0 (p = 0.01). In the test group, salivary flow significantly increased between T2 and T0 (p = 0.01), while pH alkalization was significantly greater between T4 and T0 versus control group 2 (p = 0.01) and close-to-significantly greater versus control group 1 (p = 0.06). The toothpaste with EVOO, xylitol, and betaine obtained the best outcomes in patients with gingivitis, who showed reductions in gingival bleeding and supragingival biofilm and an increase in pH at 4 months in comparison to a commercial toothpaste.

## Introduction

The oral microbiome comprises all commensal, symbiotic, and pathogenic microorganisms present in the oral cavity and is made up of viruses, fungi, protozoa, archaea, and bacteria together with their habitat or ecosystem. At least 750 species of bacteria remain in eubiosis, i.e., in dynamic balance with the host immune system and microenvironment^1,2^. Disruption of this balance generates microbiome dysbiosis, producing diseases such as caries, gingivitis, and periodontitis^3,4^. Gingivitis is a reversible inflammatory condition triggered by biofilm accumulation on the dental surface; it is characterized by reddening, edema, gingival bleeding, and the absence of periodontal insertion loss without involvement of cementum, periodontal ligament, or alveolar bone^5^. In agreement with the 2017 Workshop, a diagnosis of gingivitis is defined by ≥ 10% bleeding on probing, being considered localized when bleeding on probing is between 10 and 30% and generalized when > 30%. Gingivitis treatment includes dental prophylaxis, use of antiseptics, and appropriate oral hygiene instructions^6^.

Saliva contains 99.5% water, 0.3% proteins, and 0.2% inorganic substances, including sodium, chloride, calcium, potassium, bicarbonate, phosphate, fluoride, iodide, and magnesium^7^. It also contains proteins such as glycoproteins, mucins, immunoglobulins, lactoferrin, peroxidases, and agglutinins^8^. The average salivary flow rate is 0.33–0.55 mL/min at rest and 2 mL/min when stimulated with paraffin. Around 0.5 L of saliva is secreted daily^8^.

The salivary pH ranges from 6.2 to 7.6, with an average of pH 6.7^7^. Saliva contributes to pH maintenance via two mechanisms: the elimination of carbohydrates that could be metabolized by bacteria, and the neutralization of acidity created by food and drink or by acids from dental biofilm, through its buffer capacity^7^.

Herbal toothpastes are mostly prepared with natural ingredients^9^ and contain essential mineral salts, sodium fluoride, sodium chloride, and extracts from plants such as lemon, rosemary, chamomile, or aloe vera, among others^10^. These components act as anti-inflammatory and antibacterial agents, and various studies have recommended their use to control dental biofilm^9^. For instance, Xerostom^®^, which contains olive fruit extract, can improve the oral conditions of patients with dry mouth through its capacity to stimulate salivary secretion at rest^11^.

Few clinical trials have evaluated the effectiveness of herbal toothpastes to treat gingivitis, and only one study has assessed the in vitro antimicrobial activity of toothpaste prepared with olive fruit extract^12^. Research is needed on the effectiveness of toothpaste composed of extra-virgin olive oil (EVOO), xylitol, and betaine to reduce the formation of dental biofilm and gingivitis bleeding in comparison to conventional toothpastes. Our alternative hypothesis was that toothpaste with natural products may be more effective to improve periodontal and salivary variables in comparison to a placebo and a commercial anti-gingivitis toothpaste.

The objective of this interim study in patients with gingivitis was to determine the effects on gingival bleeding, supragingival biofilm, and salivary flow and pH of toothpaste formulated with EVOO, xylitol, and betaine in comparison to a placebo toothpaste and a commercial toothpaste indicated for gingivitis.

## Results

**Figure 1:**
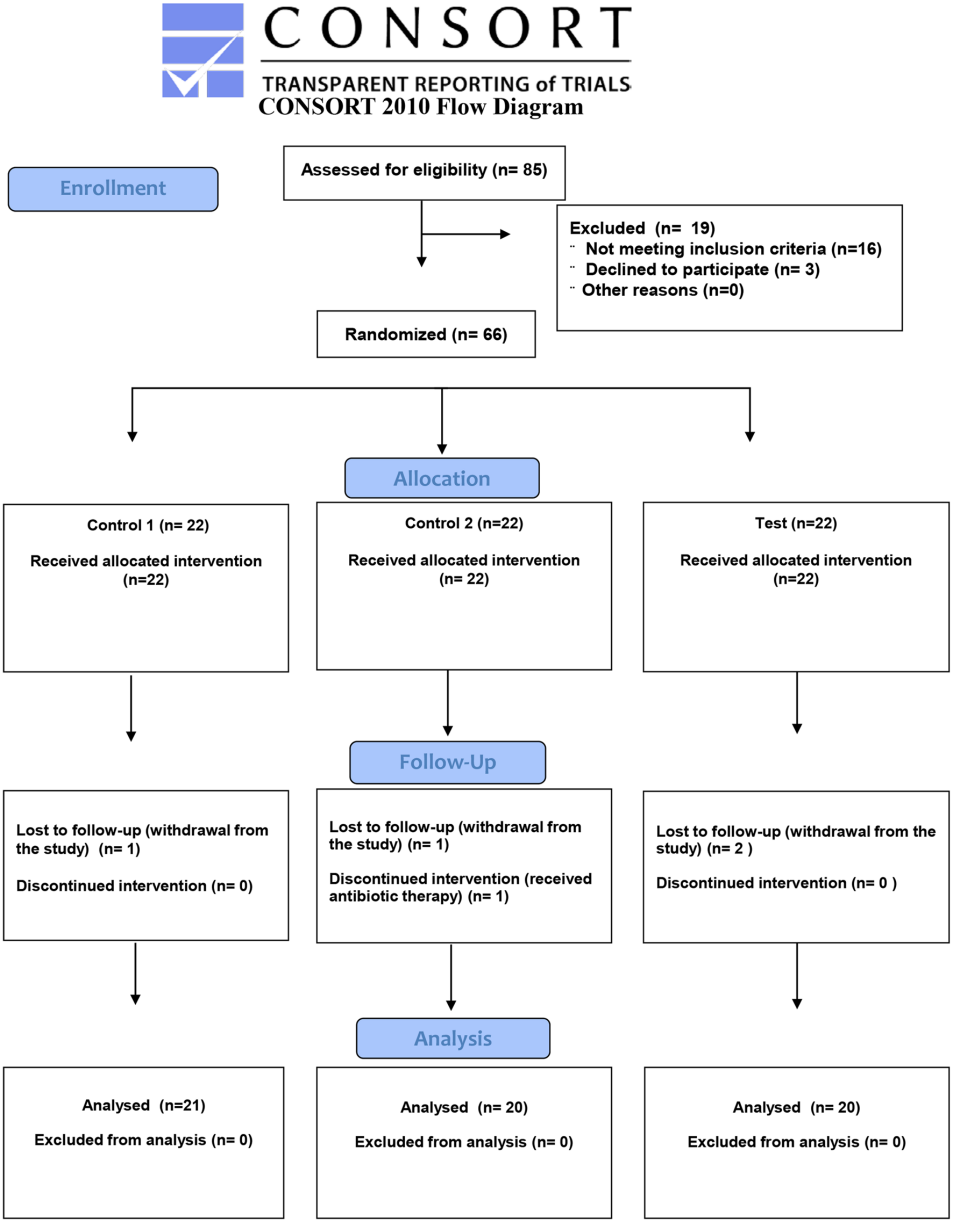
CONSORT flow diagram showing the enrolment, allocation, follow-up, and analysis of the participants in the study.

Out of 85 patients initially recruited for the study, 61 were finally included. The flowchart in Fig. 1 explains the reasons for losses. Among the 61 patients in the final study sample, 51 were examined at the Granada University School of Dentistry, 4 in the Huetor-Tajar health center, and 6 in the Loja health center. The study groups did not significantly differ in sociodemographic variables or habits, as shown in Table 1.

**Table 1:** Description of sociodemographic and habit-related variables in the study groups.

Variable	Control 1 (n = 21)	Control 2 (n = 20)	Test (n = 20)	Global *p*-value
Sex, female (%)	61.9	80.0	70.0	0.446^a^
Age (yrs.) (range)	18–65	20–66	19–60	
Age (yrs.) (mean ± sd)	31.9 ± 15.1	33.1 ± 13.6	33.3 ± 13.1	0.938^b^
Studies, University (%)	85.7	90.0	70.0	0.222^a^
Cigs./day (mean ± sd)	0.0 ± 0.0	0.3 ± 0.9	1.3 ± 3.1	0.0**7**1^b^
SDU/day (mean ± sd)	0.36 ± 0.44	0.44 ± 0.60	0.32 ± 0.33	0.686^b^
Alcohol (gr./day) (mean ± sd)	3.6 ± 4.4	3.5 ± 4.8	3.2 ± 3.3	0.946^b^

*SDU* Standard Drink Unit.

^a^Chi-square test, ^b^ANOVA.

Within-group comparisons showed a significant reduction in bleeding (*p* < 0.001) between T0 and T4 in all three groups. A significant reduction in bleeding between T2 and T4 was observed in the test group (*p* = 0.022) and control group 1 (*p* = 0.018) but not in control group 2 (*p* = 0.474). Between-group comparisons revealed a significantly greater decrease between T0 and T4 in the test group than in control group 1 (*p* = 0.050), as reported in Table 2.

**Table 2:** Bleeding on probing results over time in three study groups: baseline, 2-month, and 4-month follow-up.

Time	Control 1 (n = 21)	Control 2 (n = 20)	Test (n = 20)	Global *p*-value
Between-group comparisons^a^				
T0 (baseline) (%), mean ± sd	34 ± 12	38 ± 14	44 ± 18	0.129
T2 (%), mean ± sd	20 ± 9	20 ± 13	22 ± 11	
T4 (%), mean ± sd	17 ± 10	19 ± 13	16 ± 10	
Within-group comparisons				
Global comparison^b^, *p*-value	< 0.001	< 0.001	< 0.001	
Paired comparisons^c^, *p*-value				
T0 vs T2	< 0.001	< 0.001	< 0.001	
T0 vs T4	< 0.001	< 0.001	< 0.001	
T2 vs T4	0.018	0.474	0.022	
Variable change^a^				
T2-T0 (%), mean ± sd	− 14 ± 7	− 18 ± 12	− 22 ± 15	0.107
T4-T0 (%), mean ± sd	− 17 ± 8	− 19 ± 11	− 28 ± 18	0.042^d^
T4-T2 (%), mean ± sd	− 3 ± 7	− 1 ± 6	− 6 ± 10	0.177

^a^ANOVA was used for between-groups and variable change comparisons (three groups). If *p*-value < 0.05, post-hoc paired comparisons (with Bonferroni’s method) were also performed (depicted in footnote d).

^b^For intra-group comparisons (three times), Friedman’s test was used.

^c^Student’s T-test for paired samples.

^d^Post-hoc paired comparisons for independent groups: Significant difference between Test and Control 1 (− 10% *p* = 0.050; 95%-CI = − 20, − 1).

Table 3 shows the results regarding plaque index. Within-group comparisons showed a statistically significant reduction in plaque level (*p* < 0.001) in all three groups. This reduction was present in all times when paired comparison were performed. Between-group comparisons for plaque levels showed a significantly greater decrease between T0 and T2 in the test group than in control group 1 (*p* = 0.047). A greater reduction was also observed between T0 and T4 in the test group compared to both control group 1 (*p* = 0.020) and control group 2 (*p* = 0.030).

**Table 3:** Plaque index results over time in three study groups: baseline, 2-month, and 4-month follow-up.

Time	Control 1 (n = 21)	Control 2 (n = 20)	Test (n = 20)	Global *p*-value
Between-group comparisons^a^				
T0 (baseline) (%), mean ± sd	38 ± 13	41 ± 14	50 ± 20	0.076
T2 (%), mean ± sd	24 ± 13	25 ± 15	26 ± 14	
T4 (%), mean ± sd	19 ± 12	21 ± 13	19 ± 11	
Within-group comparisons				
Global comparison^b^, *p*-value	< 0.001	< 0.001	< 0.001	
Paired comparisons^c^, *p*-value				
T0 *vs* T2	< 0.001	< 0.001	< 0.001	
T0 *vs* T4	< 0.001	< 0.001	< 0.001	
T2 *vs* T4	0.014	0.006	0.007	
Variable change^a^				
T2-T0 (%), mean ± sd	− 14 ± 11	− 16 ± 12	− 24 ± 15	0.033^d^
T4-T0 (%), mean ± sd	− 19 ± 9	− 20 ± 11	− 31 ± 18	0.010^e^
T4-T2 (%), mean ± sd	− 5 ± 9	− 4 ± 7	− 7 ± 11	0.720

^a^ANOVA was used for between-groups and variable change comparisons (three groups). If *p*-value < 0.05, post-hoc paired comparisons (with Bonferroni’s method) were also performed (depicted in footnotes d and e).

^b^For intra-group comparisons (three times), Friedman’s test was used.

^c^Student’s T-test for paired samples.

^d^post-hoc paired comparisons for independent groups: Significant difference between Test and Control 1 (− 10%, *p* = 0.047; 95%-CI = − 19, − 1).

^e^Post-hoc paired comparisons for independent groups: Significant difference between Test and Control 1 (− 11%, *p* = 0.020; 95%-CI = − 21, − 1), and between Test and Control 2 (− 11% *p* = 0.030; 95%-CI = − 21, − 1).

No significant differences in salivary flow were observed among the three groups at any time point, as shown in Table 4. However, a significant increase in salivary flow (*p* = 0.017) was detected between T0 and T2 in the test group. There appeared to be a trend for the salivary pH to decrease in the control groups and increase in the test group at T4. Between T4 and T0, salivary pH changes in the test group (increased pH) significantly differed (*p* = 0.01) from those in control group 2 (decreased pH) and were close-to-significance (*p* = 0.06) from those in control group 1.

**Table 4:** Salivary flow and pH results over time in three study groups: baseline, 2-month, and 4-month follow-up.

Time	Control 1 (n = 21)	Control 2 (n = 20)	Test (n = 20)	Global *p*-value
Sialometry (ml./min.)				
Between-group comparisons^a^				
T0 (baseline) (%), mean ± sd	0.57 ± 0.34	0.40 ± 0.23	0.46 ± 0.42	0.272
T2 (%), mean ± sd	0.63 ± 0.40	0.38 ± 0.28	0.59 ± 0.39	
T4 (%), mean ± sd	0.57 ± 0.42	0.44 ± 0.33	0.49 ± 0.29	
Within-group comparisons				
Global comparison^b^, *p*-value	0.273	0.988	0.026	
Paired comparisons^c^, *p*-value				
T0 *vs* T2	0.315	0.612	0.017	
T0 *vs* T4	0.924	0.433	0.602	
T2 *vs* T4	0.392	0.284	0.078	
Variable change^a^				
T2-T0 (%), mean ± sd	0.06 ± 0.29	− 0.02 ± 0.15	0.13 ± 0.22	0.132
T4-T0 (%), mean ± sd	0.00 ± 0.25	0.04 ± 0.21	0.03 ± 0.22	0.824
T4-T2 (%), mean ± sd	− 0.06 ± 0.36	0.06 ± 0.22	− 0.10 ± 0.25	0.196
pH				
Between-group comparisons^a^				
T0 (baseline), mean ± sd	7.27 ± 0.46	7.20 ± 0.62	7.38 ± 0.57	0.584
T4, mean ± sd	7.06 ± 0.48	6.81 ± 0.49	7.43 ± 0.62	
Within-group comparisons^c^	0.016	0.003	0.690	
Variable change^a^				
T4-T0 (%), mean ± sd	− 0.21 ± 0.38	− 0.39 ± 0.53	0.05 ± 0.50	0.016^d^

^a^ANOVA was used for between-groups and variable change comparisons (three groups). If *p*-value < 0.05, post-hoc paired comparisons (with Bonferroni's method) were also performed (depicted in footnote d).

^b^For intra-group comparisons (three times), Friedman's test was used.

^c^Student's T-test for paired samples.

^d^Post-hoc paired comparisons for independent groups: Significant difference between Test and Control 2 (0.44, *p* = 0.014; 95%-CI = 0.07, 0.81).

## Discussion

The main finding of this study was a reduction in gingival bleeding among patients with gingivitis who used a toothpaste with EVOO as main ingredient compared with those using a placebo toothpaste (control group 1). This result can be attributed to multiple biological mechanisms, with a potential role played by the combined effect of the phenolic compounds and other minority compounds present in olive oil, especially in young EVOO, as discussed below.

Hydroxytyrosol, obtained by oleuropein hydrolysis, is a phenolic compound present in olive leaves and fruit that exerts potent antioxidant, anti-inflammatory, and antibacterial effects^13^. Bertelli et al. recently demonstrated a promising anti-inflammatory effect of this product by reducing the synthesis of proinflammatory cytokines TNF-α, IL-1β, IL-6, and cyclooxygenase-2 (COX-2)^14,15^.

Oleocanthal is a phenolic compound obtained in just-pressed EVOO, and its structure was found in an in vitro study to be similar to that of ibuprofen (non-steroidal anti-inflammatory drug) and to produce a comparable stinging sensation in the throat^16^, as reported by several patients in the present study. Oleocanthal has a greater capacity to inhibit both cyclooxygenases (COX-1 and COX-2) in comparison to ibuprofen at the same concentrations^16^. Other in vitro studies found that oleocanthal can reduce the production of proinflammatory cytokines IL-1β, TNF-α, and nitric oxide^14,17^.

Oleacein, another phenolic compound in EVOO, structurally derives from glucoside oleuropein^18^ and is known to exert multiple anti-inflammatory actions at different levels. It reduces the secretion by human neutrophils of myeloperoxidases, proinflammatory mediators that can exacerbate tissue damage^19^. It inhibits the expression of adhesion molecules VCAM-1, ICAM-1, and E-selectin, thereby reducing immune cell migration^20^, and it favors the expression by macrophages of CD 163 receptor (related to inflammatory reaction regulation phase) and IL-10 (anti-inflammatory cytokine)^21^.

The test toothpaste also contains xylitol, which has been found to inhibit the synthesis of TNF-α and IL-1β induced by lipopolysaccharides from *Porphyromonas gingivalis* through NF-κB pathway activation^22^. The EVOO phenols and xylitol in the test toothpaste may therefore have combined effects on gingival bleeding.

In our study, dental biofilm reduction was greater with the test toothpaste than with the control toothpastes, which may also be related to the antibacterial effect of *olea europaea* described in in vitro studies^23^.

Karygianni et al. conducted an in vitro study to determine the antibacterial effect of maslinic acid, hydroxytyrosol, oleocanthal, and oleacein against eight oral bacterial species (*Streptococcus mutans, S. sobrinus, S. oralis, Enterococcus faecalis, P. gingivalis, Parvimonas micra, Fusobacterium nucleatum*) and *Candida albicans*^24^. Maslinic acid is a natural pentacyclic triterpenoid and damages the cell membrane of Gram-positive and Gram-negative bacteria. *Lactobacillus plantarum* can hydrolyze and transform oleuropein into hydroxytyrosol, which is highly effective against anaerobic Gram-negative bacteria such as *P.gingivalis*^24^*.*

Other documented effects of xylitol include the reduction of bacterial plaque by decreasing the adhesion of *S. mutans*, the main primary colonizer of dental biofilm^25^. Burt et al. reported that xylitol cannot be metabolized by the microorganisms in dental biofilm and inhibits the growth of *S. mutans* through inanition^26^. In addition, xylitol is transformed into xylitol-5-phosphate by phosphoenolpyruvate, resulting in the production of intracellular vacuoles and cell membrane degradation. *S. mutans* dephosphorylates xylitol-5-phosphate, and when this sugar molecule is expelled from the cell, bacteria generate energy expenditure in the absence of any energy supply^27^.

Other main component of this toothpaste is betaine (trimethylglycine.). Animal studies showed that betaine suppressed the activity of NF-κB and a wide range of inflammation-related genes, including TNF-α, VCAM-1 and ICAM-1^28^. Betaine inhibits NF-κB through the suppression of two important activators, mitogen-activated protein kinases (MAPKs) and NF-κB-inducing kinase/Inhibitory-κB kinase (NIK/IKK)^28,29^.

Salivary flow was not significantly increased by the test toothpaste in comparison to the control toothpastes. However, it significantly increased in the test group alone over the first 2 months (*p* = 0.017) (T2), and a higher flow was observed between baseline and 4 months, although statistical significance was not reached.

The test toothpaste increased salivary pH to a slightly alkaline pH with a mean of 7.5, being more favorable for oral eubiosis^30^. The pH value is important for preserving correct cell biochemistry and tissue homeostasis, and it is low at sites with inflammation or cell destruction^31^. The response of macrophages to an acid pH environment was found to involve the activation of inflammasome NLRP3, leading to the secretion of proinflammatory cytokines IL-1β, IL-18, and IL-33^32^. In the same study, alkalinization of the environment was reported to inhibit the activation of proinflammatory system NLRP3, leading the authors to propose environment pH modulation as a potential novel anti-inflammatory therapy^32^.

It was recently demonstrated that periodontal pathogens grow in a moderately acid environment, e.g., pH of 5.0–7 for *P. intermedia*, pH of 5.5–7 for *F. nucleatum*, and pH of 6.5–7 for *P. gingivalis*. Also, the salivary pH of patients with periodontitis became more alkaline after scaling and root planning^30^. A recent study described both salivary flow and pH as markers of periodontitis severity, which was correlated with low pH values (6.25 in grade IV periodontitis) and low salivary flow (0.28 mL/min); conversely, pH and flow values were significantly increased in patients with severe periodontitis after 3 months of periodontal treatment (hygienization)^33^.

Study limitations include the relatively small sample size, although it was estimated to offer sufficient statistical power, and some statistically significant differences. On the other hand, this interim study is the first multicenter, parallel-group, double-blind, placebo-controlled randomized clinical trial to evaluate clinical changes in gingival bleeding, dental biofilm, salivary flow, and saliva pH after using a toothpaste with a natural product (EVOO) as the main ingredient.

Superior clinical outcomes were obtained at 4 months in patients with gingivitis who brushed their teeth three times daily with the toothpaste containing EVOO, xylitol, and betaine in comparison to a placebo toothpaste and commercial anti-gingivitis toothpaste, including a reduction in gingival bleeding as well as an increase in salivary pH, contributing to oral eubiosis. Further research is warranted to verify these findings in wider samples.

## Methods

This multicenter, parallel-group, double-blind, placebo-controlled randomized clinical trial recruited patients from two primary healthcare centers (Loja and Huetor Tajar) in the province of Granada (Southern Spain) and from the Clinic of the School of Dentistry of Granada University from July 2021 until September 2022. The study complied with the principles of the 2013 revision of the Declaration of Helsinki and was approved by the Biomedical Research Ethics Committee of Andalusia (ref.2184-N-20.). Written informed consent was obtained from all participant at the time of being enrolled in the study. The trial is registered on the ClinicalTrials.gov webpage (Study on the effects of a toothpaste in the microbiome and clinical parameters in patients with oral dysbiosis. ref. NCT05463484, 19/07/2022) and reported in accordance with CONSORT guidelines^34^.

Inclusion criteria were age between 18 and 70 years, the presence of at least 20 teeth without counting third molars, a diagnosis of gingivitis (index of bleeding on probing ≥ 10%) according to the 2017 World Workshop^6^, the signing of informed consent^35^, and explicit commitment to a manual toothbrushing regimen of three times a day for 4 min. Exclusion criteria were the presence of periodontitis, orthodontic appliances, removable partial prostheses, soft or hard tissue tumors of the oral cavity, > 5 caries lesions requiring immediate restoration, or allergies to oral hygiene products or specific ingredient(s) of the toothpastes under study, the receipt of antibiotic therapy during the previous month, receipt of dental prophylaxis during the previous 2 weeks, and being pregnant or breastfeeding^35^.

Patients were randomly assigned by Mucosal Innovations S.L to one of the following three toothpaste groups using a random number generator (http://www.random.org): *Test Group*: toothpaste with EVOO extract, xylitol, betaine, water, hydrated silica, glycerol, sodium monofluorophosphate, titanium dioxide, sodium benzoate, aroma, potassium sorbate, and stevia (FDA 510(k) ref. number K142657), *Control Group 1*: placebo toothpaste with the same ingredients as the experimental toothpaste but without EVOO, xylitol, or betaine. *Control Group 2*: commercial anti-gingivitis toothpaste, containing water, hydrolyzed hydrogenated starch, hydrated silica, zinc citrate, sodium lauryl sulfate, aroma, cellulose gum, sodium fluoride, sodium saccharin, tocopheryl acetate, and titanium dioxide.

The researcher gave the corresponding toothpaste to each patient, with both being blinded to the type of toothpaste, which was labeled with a code. Patients were instructed to brush their teeth with the assigned toothpaste for 4 min three times a day for 4 months and to use no other oral hygiene product. Clinical evaluations were performed at baseline (T0), 2 months (T2), and 4 months (T4) with the exception of the pH measurement (at T0 and T4). Patients were asked not to eat or smoke and to drink only water during the four hours before appointments^35^.

Data were gathered on patient age, sex, schooling level (compulsory schooling, high school, vocational training, and university education), tobacco consumption (cigarettes/day), alcohol consumption (standard drink units [SDUs]/day), and diabetes (yes/no). Scores were also collected for the plaque index proposed by Tonetti^36^ and the gingival bleeding index described by Ainamo and Bay^37^, using a PCPUNC15 periodontal probe (Hu-friedy, Chicago, IL, EEUU) at six sites per tooth (mesio-vestibular, vestibular, disto-vestibular, disto-lingual, lingual, mesio-lingual). A sample of non-stimulated salivary flow was gathered following Navazesh^38^, with the patient in physiological resting position, mouth closed and head tilted slightly backwards, without speaking, and spitting into a container every 60 s for 5 min. The total volume was collected in a syringe and divided by five to calculate the saliva flow (in mL) per minute.

Salivary pH was determined in saliva gathered at rest at T0 and T4 using PH 60 DHS equipment (XS Instruments, Carpi [MO], Italy) and an electrode (ref. H-238150) with diameter of 6 mm and minimum immersion of 15 mm that scales the pH from 0 to 14.

### Statistical analysis

Sample Power 2.0 (SPSS Inc., Chicago, IL) was used to calculate the sample size required to detect with 80% power and 5% alpha error, using the Student’s t-test for independent samples, a standardized difference of 0.9 on Cohen’s scale^39^ among the groups in study variables (gingivitis, plaque, salivary flow, and pH). A minimum sample size of 20 patients per group was estimated.

For *p*-value calculations, briefly, we used different descriptive and analytic methods, depending on the type of variable (qualitative or quantitative). These methods are described in table footnotes. For quantitative variables in inter-group comparisons, we used ANOVA with Bonferroni's method for post-hoc paired comparisons when the global *p*-value was significant, i.e., p < 0.005. For intra-group comparisons Friedman's test was applied, and Student's T-test for paired comparisons when Friedman's test was significant.

## Data Availability

The datasets analyzed during the current study are available from the corresponding author on reasonable request, considering the privacy and data protection.
